# Fe_3_O_4_-PEI Nanocomposites for Magnetic Harvesting of *Chlorella vulgaris*, *Chlorella ellipsoidea*, *Microcystis aeruginosa*, and *Auxenochlorella protothecoides*

**DOI:** 10.3390/nano12111786

**Published:** 2022-05-24

**Authors:** Kristína Gerulová, Alexandra Kucmanová, Zuzana Sanny, Zuzana Garaiová, Eugen Seiler, Mária Čaplovičová, Ľubomír Čaplovič, Marián Palcut

**Affiliations:** 1 Institute of Integrated Safety, Faculty of Materials Science and Technology, Slovak University of Technology, J. Bottu 25, 917 24 Trnava, Slovakia; kristina.gerulova@stuba.sk (K.G.); alexandra.kucmanova@stuba.sk (A.K.); zuzana.sanny@stuba.sk (Z.S.); 2 Department of Nuclear Physics and Biophysics, Faculty of Mathematics, Physics and Informatics, Comenius University, Mlynská Dolina F1, 842 48 Bratislava, Slovakia; zuzana.garaiova@fmph.uniba.sk; 3 Institute of Electrical Engineering, Slovak Academy of Sciences, Dúbravská Cesta 9, 841 04 Bratislava, Slovakia; eugen.seiler@savba.sk; 4 Centre for Nanodiagnostics of Materials, Faculty of Materials Science and Technology, Slovak University of Technology, Vazovova 5, 812 43 Bratislava, Slovakia; maria.caplovicova@stuba.sk; 5 Institute of Materials Science, Faculty of Materials Science and Technology, Slovak University of Technology, J. Bottu 25, 917 24 Trnava, Slovakia; lubomir.caplovic@stuba.sk

**Keywords:** harvesting, Fe_3_O_4_, magnetite, nanoparticles, polyethyleneimine

## Abstract

Magnetic separation of microalgae using magnetite is a promising harvesting method as it is fast, reliable, low cost, energy-efficient, and environmentally friendly. In the present work, magnetic harvesting of three green algae (*Chlorella vulgaris, Chlorella ellipsoidea,* and *Auxenochlorella protothecoides*) and one cyanobacteria (*Microcystis aeruginosa*) has been studied. The biomass was flushed with clean air using a 0.22 μm filter and fed CO_2_ for accelerated growth and faster reach of the exponential growth phase. The microalgae were harvested with magnetite nanoparticles. The nanoparticles were prepared by controlled co-precipitation of Fe^2+^ and Fe^3+^ cations in ammonia at room temperature. Subsequently, the prepared Fe_3_O_4_ nanoparticles were coated with polyethyleneimine (PEI). The prepared materials were characterized by high-resolution transmission electron microscopy, X-ray diffraction, magnetometry, and zeta potential measurements. The prepared nanomaterials were used for magnetic harvesting of microalgae. The highest harvesting efficiencies were found for PEI-coated Fe_3_O_4_. The efficiency was pH-dependent. Higher harvesting efficiencies, up to 99%, were obtained in acidic solutions. The results show that magnetic harvesting can be significantly enhanced by PEI coating, as it increases the positive electrical charge of the nanoparticles. Most importantly, the flocculants can be prepared at room temperature, thereby reducing the production costs.

## 1. Introduction

The consumption of conventional fossil fuels should be reduced as the reserves of raw materials are being depleted. High expectations are placed on the production of biofuels [1,2]. There has been a growing interest in microalgae exploitation over the past decade [2]. The microalgae are important single-cell photosynthetic microorganisms that are regarded as potential biomaterial sources for biofuel feedstock and nutrition [3,4]. The microalgal biofuel production represents a more environmentally friendly alternative to first-generation biofuels [5]. The microalgal production systems do not need fertile soils. They can be grown in marginal areas such as on non-arable lands or potentially in the ocean, thereby reducing competition for agricultural land and freshwater with food crops [6,7]. The microalgal production systems do not directly compete with the food chain. Furthermore, they can be used to convert CO_2_ to oxygen or for wastewater treatment [8,9,10,11].

Microalgae constitute a diverse group of single-cell photosynthetic organisms that include a wide range of eukaryotic algae and cyanobacteria [2]. Microalgae contain high-value nutrients [4]. Depending on species, the microalgae may produce several important lipids, and other oils [12,13,14]. Microalgae strains with high oil production capabilities are required for efficient biodiesel production [3,4]. Microalgae and cyanobacteria have a considerably higher oil production rate compared to conventional crops [15]. *Chlorella* species contain approximately 30% lipids (dry mass) [16,17]. *Microcystis aeruginosa* is a cyanobacteria (blue green algae) commonly observed in still waters (lakes and reservoirs), where it contributes to the development of eutrophication and bloom formation [18,19]. Although cyanobacteria are not eukaryotic phototrophs, as green algae are, they have high productivity and vast biomass [20,21]. *Microcystis aeruginosa* has a high lipid content [21]. As such, it is used for biofuel production. Furthermore, *Microcystis aeruginosa* can also be used in the sequestration of CO_2_ from the atmosphere [22,23].

Several sequential steps are involved in microalgal biodiesel production, including cell cultivation, harvesting, extraction of lipids, and fatty acid methyl ester generation. [24]. One of the problems that limits the use of microalgal biorefineries is the harvesting process [24,25]. It is more demanding compared to crop harvesting. The cost of the harvesting step can reach 20–30% of the total costs of algal-based biofuels’ production [26,27,28]. During microalgae harvesting, the water content is gradually removed from the microalgae culture medium through several subsequent techniques to concentrate biomass [29,30]. The choice of a suitable harvesting method is influenced by, for example, algae species (cell size, viability, and density, possible cell damage, strain properties, sedimentation rate, salt concentration) [29,31,32] and reuse of culture medium. It should be cheap and nontoxic when applied on a large scale. The suitability of the harvesting method depends on its energy demands, duration, financial needs, and finally, its environmental friendliness [33,34].

Several techniques have been developed for microalgae harvesting, including magnetic separation, centrifugation, flocculation, filtration, sedimentation, flotation, and electrophoresis [35]. Magnetic nanoparticles can be used to capture living algal cells rapidly and effectively, followed by low-energy magnetic isolation [36]. The magnetic separation using magnetic nanoparticles is regarded as one of the most promising methods. It is fast, energy efficient, low cost, environmentally friendly, scalable, and low contamination [1,37].

Magnetic nanoparticles are versatile materials with multiple applications [38,39]. They can be produced by several methods, including microemulsion [40,41], hydrothermal synthesis [42,43], thermal decomposition method [44,45], pyrolysis [46,47], sol-gel synthesis [48,49], and co-precipitation with bases [50,51]. Among these methods, the co-precipitation is the most used process [52]. It is easy to operate, and it can produce large volumes of nanoparticles. The co-precipitation is used to synthesize iron oxides and other ferrites [53,54]. A Fe^3+^/Fe^2+^ molar ratio of 2 is required for the synthesis of Fe_3_O_4_ nanoparticles. The formation of Fe_3_O_4_ by co-precipitation of Fe^3+^ and Fe^2+^ can be expressed by the following reaction [52,55]:

Fe^2+^ + 2Fe^3+^ + 8OH^−^ → Fe_3_O_4_ + 4H_2_O (1)

In the Equation (1), a dropwise addition of ammonia is required to increase the pH of the solution. The co-precipitation is typically performed in an inert atmosphere (N_2_ or Ar) to avoid oxidation of Fe_3_O_4_ to Fe_2_O_3_. The reaction yield can be increased by vigorous stirring. The grain size and magnetic properties of the nanoparticles can be adjusted by controlling the reaction conditions [56,57]. Furthermore, the addition of different oxidizing and chelating agents, e.g., surfactants, saccharides, and polymers, is possible, which can influence the characteristic properties of the prepared nanoparticles [58].

A bare magnetite was used for the harvesting of *Chlorella pyrenoidosa* and *C. minutissima* [59], *Nannochloropsis maritime* [60], *Scenedesmus obliquus* [61], and *Chlorella vulgaris* [62,63,64]. The uncoated magnetic nanoparticles (NPs) were also studied in the harvesting of *Microcystis aeruginosa* [65]. Nevertheless, the electrostatic attraction between magnetite and algae species is not optimal. The functionalization of magnetite is usually necessary for higher harvesting efficiency or reactivation and for magnetite dosage reduction [66]. Polyethyleneimine (PEI) is a cationic polymer with repeating CH_2_CH_2_NH units. It can be easily protonated in acidic media. If adsorbed on the magnetite nanoparticles, it can functionalize them and modify their electrical charge [66]. PEI-coated nanoparticles were studied, for example, by Ge et al. for *Scenedesmus dimorphus* [67], Wang et al. [68] and Yang et al. [69] for *Microcystis aeruginosa*, and Hu et al. for *Chlorella ellipsoidea* [70].

The algal biomass increases with the culture time [71]. Hu et al. showed that the maximum harvesting efficiency could be obtained on days 14–18, i.e., when the biomass reaches the peak value [70,72]. As the algal biomass increases, the probability of interactions between the cells and nanoparticles also increases. The growth stage of algae has an influence on the lipid content and surface characteristics of the algal cells [33,73]. The number of functional groups on algal cells increases during the exponential growth phase, which enhances the adsorption capacity of the surface-functionalized magnetic particles [74]. The PEI-coated iron oxide nanoparticles can also be used to remove extracellular organic matter of the cells via charge neutralization. The harvesting and extracellular organic matter removal can be conducted simultaneously [68,75]. The nanoparticles can be removed by acid-base treatment and ultrasonication from the attached cells and re-used for further harvesting, which makes the process economical [76].

The above-mentioned studies of algae sorption to magnetite have been performed with magnetite nanoparticles synthetized at high temperatures (typically 80 °C). In the present study, we have prepared the iron oxide nanoparticles at room temperature and coated them with polyethyleneimine. The lower temperature was used to decrease the energy consumption of the process. The results show that the synthesis of PEI-coated Fe_3_O_4_ at 20 °C is feasible. The nanoparticles have comparable characteristics (particle size, microstructure, harvesting efficiency) to NPs prepared at 80 °C. The harvesting efficiencies can be significantly enhanced by PEI coating, as the polymer increases the positive electrical charge of the nanoparticles. In the present work, we study the magnetic harvesting of three green algae (*Chlorella vulgaris, Chlorella ellipsoidea,* and *Auxenochlorella protothecoides*) and one cyanobacteria (*Microcystis aeruginosa*) to explore the applicability of the process on several microorganisms. The results show that the magnetic harvesting with the nanoparticles synthesized at room temperature is applicable to both eukaryotic algae and cyanobacteria, making the process attractive for industrial use.

## 2. Materials and Methods

### 2.1. Magnetite Nanoparticles’ Synthesis

Magnetite nanoparticles (Fe_3_O_4_ NP) were synthesized by controlled co-precipitation of Fe^2+^ and Fe^3+^ chlorides in NH_4_OH. We used a previously reported method [70], however, the experiments were conducted at room temperature (20 °C) instead of 80 °C to decrease the production costs. The distilled water was de-oxygenated prior to the experiment by purging with flowing N_2_ for 30 min. For one dose of Fe_3_O_4_ NP, 1.98 g of FeCl_2_.4H_2_O and 5.4 g of FeCl_3_.6H_2_O were placed in a three-neck flask vessel and dissolved in 200 mL of deoxygenated distilled water. The resulting aqueous solution was vigorously stirred and constantly purged with flowing N_2_. After a dropwise addition of 20 mL of NH_4_OH (25 wt.%) and continuous stirring for 30 min, Fe_3_O_4_ NPs were precipitated. The concentration of the prepared Fe_3_O_4_ NP was 0.05 mol L^−1^. The nanoparticles were sedimented for 1 h. Subsequently, the precipitate was washed with distilled water. The decantation process was repeated three times. The sedimentation was aided by using a neodymium magnet.

### 2.2. PEI Coating Procedure

Decantated NPs from the co-precipitation were admixed with phosphate buffer (pH 7.3). Subsequently, polyethyleneimine (PEI) solution (1.2 kDa, 50% (*w*/*v*) in H_2_O, Sigma Aldrich, Bratislava, Slovakia) was added. The volume ratio of PEI:Fe_3_O_4_ was 9:1. The mixture was stirred at 150 rpm for 1 h, at laboratory temperature (20 °C). The prepared nanocomposites (NCs) were washed three times with distilled water, and stored in a sealed glass bottle for further use. All chemicals were analytical grade and were used without prior purification.

### 2.3. Transmission Electron Microscopy and X-ray Diffraction

The microstructure and particle size of Fe_3_O_4_ NPs and NCs were studied by a double-corrected, high-resolution scanning transmission electron microscope, JEOL JEM ARM 200cF (STEM resolution 0.78 Å, TEM resolution 1.1 Å, Tokyo, Japan). The samples for TEM observation were prepared by dropping the aqueous Fe_3_O_4_ solution onto a carbon layer-covered copper grid and air-dried. The particle size distribution was estimated from TEM images using ImageJ, a Java-based image processing program. An electron diffraction analysis was also employed to study the phase constitution of the prepared materials.

A PANalytical Empyrean X-ray diffractometer (XRD, Malvern Panalytical Ltd., Malvern, UK) was used to study the phase constitution of prepared nanoparticles and nanocomposites. The diffractometer was working with a CoKα1,2 radiation source and operating at 40 kV and 30 mA. The diffraction patterns were recorded at room temperature using Bragg–Brentano geometry. The measurements were carried out at 20° to 120° (2 Theta), with a step size of 0.02° and counting time of 98 s per step.

### 2.4. Magnetic Properties

The magnetic properties of the prepared and air-dried Fe_3_O_4_ NPs and NCs were studied by DC magnetometry. A plastic container with approximately 0.015 g of densely packed nanoparticle powder was placed in a vibrating sample magnetometer. The magnetic moment was measured at room temperature by generating an external homogeneous magnetic field with induction B, which was applied perpendicularly to the pre-dried sample. Magnetization loops were recorded between B = −2 T and B = +2 T (magnetic field strength between −20,000 and +20,000 Oe). A constant sweeping rate was used in all measurements.

### 2.5. Zeta Potential

Zeta potential measurements of the algae species, NPs, and NCs were performed using a Zetasizer Nano-ZS (Malvern, UK). The instrument uses a He-Ne laser with a wavelength of 633 nm and M3-PALS technology. The electrophoretic mobilities were converted into zeta potentials via the Henry equation in the Smoluchowski approximation [77]. The stock solution was 46 mg mL^−1^ for noncoated magnetite and 109 mg mL^−1^ for PEI-coated magnetite. The samples for electrokinetic potential measurements were prepared by micro-pipetting 2 μL of magnetic nanoparticles from the stock solution and mixing them with 998 μL of working buffer. In parallel, 100 μL of algae stock solution (concentration approximately 0.8 g L^−1^ DCW, in culture medium) was diluted in 900 μL of working buffer. Na-phosphate buffer (10 mmol L^−1^, NaH_2_PO_4_ and Na_2_HPO_4_) was used as a working buffer. The pH of 2.4–9.0 was adjusted by adding small aliquots of 1 mol L^−1^ of either HCl or NaOH. The tested solutions were measured in a disposable clear folded capillary zeta cell (DTS1070; Malvern, UK) at 25 °C. Values are reported as an average from 3 consecutive measurements, following an automatic measurement duration of 10–30 runs.

### 2.6. Microalgae Strains and Cultivation

*Chlorella vulgaris* (SAG 211-11b), *Chlorella ellipsoidea* (SAG 2111), *Microcystis aeruginosa* (SAG 46.80), and *Auxenochlorella protothecoides* (SAG 33.80) were obtained as sterile cultures from the algae collection of the University of Göttingen, Germany (SAG—Sammlung von Algenkulturen der Universität Göttingen). The biomass cultivation for the harvesting experiment was carried out in 1 L Erlenmeyer flasks using standard BG 11 cultivation medium. The biomass was illuminated at 2000 lx at 25 °C, with a light/dark cycle of 16/8 h. The biomass was flushed with clean air using a 0.22 μm filter. The algae were fed CO_2_ from the air to accelerate growth and accelerate reaching the exponential growth phase. The algae concentration (g L^−1^) was calculated using the calibration curve of the known optical density at 680 nm using a Genesys 8 spectrophotometer according to the dry cell weights determined gravimetrically after drying to constant weight at 110 °C.

### 2.7. Magnetite Harvesting Procedure

A known amount of either uncoated magnetite or PEI-coated magnetite was added to 50 mL of algae suspension. The flask was shaken manually for 90 s and subsequently placed on a permanent NdFeB block magnet (permanent magnetization 1.22–1.30 T, Magsy Ltd., Los Angeles, CA, USA) for 10 min. The optical densities of the remaining supernatant were measured at 680 nm using a Genesys 8 spectrophotometer. A harvesting efficiency, *R* (%), was calculated according to the following equation:

(2) $$R=\frac{\left(C_{0}-C_{e}\;\right)}{C_{0}}\times 100\;\left(\%\right),$$

In Equation (2), *C*_0_ is the initial concentration of the algae suspension (g L^−1^) and *C_e_* is the concentration of algae in the supernatant after harvesting (g L^−1^).

### 2.8. Adsorption Experiments

Adsorption experiments were carried out with approximately 2-week-old microalgae. An optimal pH of 7.0 and dose of 5–30 mg of noncoated or PEI-coated magnetite were used at a constant temperature (25 °C), volume of algae 50 mL, reaction time 90 s, and stirring speed of approximately 120 r min^−1^. Two different isotherm models, Langmuir and Freundlich, have been tested (Table 1).

The algae cells with adsorbed Fe_3_O_4_-PEI NCs were transferred to a slide (base glass) and inspected with a light microscope, Carl Zeiss Jenavert.

### 2.9. Statistical Analysis

The algal experiments were performed at room temperature (20 ± 2 °C). We used triplicate sampling and testing. The results in this paper are presented as mean values calculated from three experiments. The standard deviation was also calculated from three independent measurements. The triplicated datasets of each experiment were analyzed statistically using one-way analysis of variance at a significance level of 0.05. The statistical analysis was integrated in the statistical software OriginPro 8.5.

## 3. Results

### 3.1. Characterization of Prepared Fe_3_O_4_ and Fe_3_O_4_-PEI NPs

In this paper, we investigated the effect of the synthesis temperature on the microstructure and magnetic properties of PEI-coated Fe_3_O_4_ NPs. Originally, a relatively high temperature (80 °C) was applied for the co-precipitation of Fe^3+^ and Fe^2+^ in alkaline medium [70]. The high temperature was used to accelerate the chemical reaction. However, it was later reported that the synthesis temperature can be lowered and used to control the size of prepared Fe_3_O_4_ NPs [78,79].

**Figure 1:**
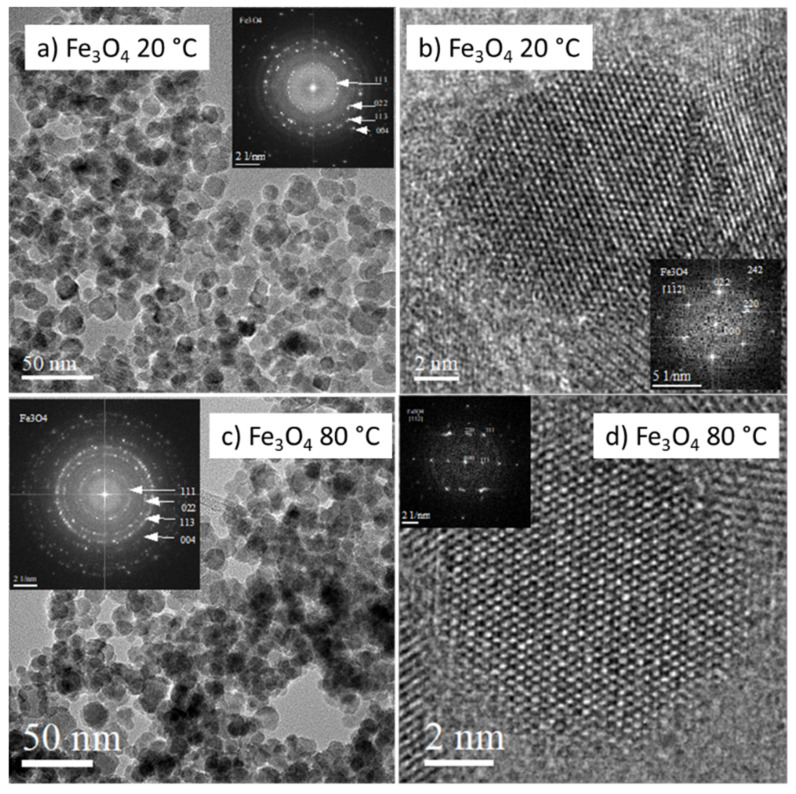
Microstructure of naked Fe_3_O_4_ NPs prepared at 20 °C (**a**,**b**) and 80 °C (**c**,**d**), respectively. Low-magnification TEM images of NPs with relevant SAED pattern in insets (**a**,**c**). HRTEM/ARTEM images of individual NPs with relevant FFT patterns in insets (**b**,**d**).

**Figure 2:**
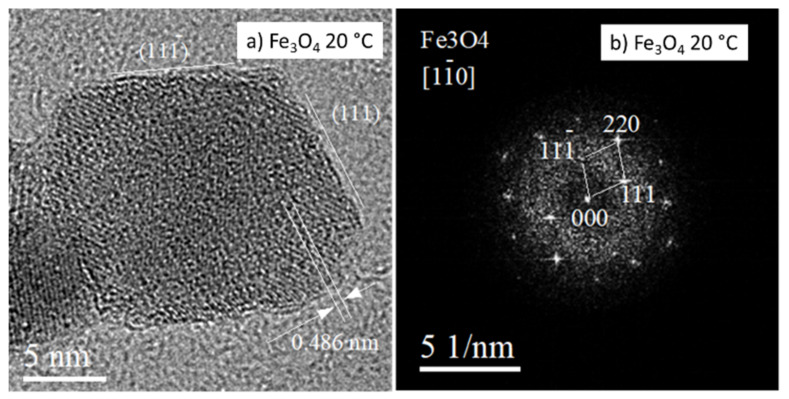
HRTEM detail of naked NP produced at 20 °C (**a**). Relevant FFT pattern confirming octahedral morphology of NP faceted predominantly by {111}-type planes (**b**).

The microstructure of magnetite Fe_3_O_4_ NPs prepared at 20 and 80 °C as recorded by TEM imaging is provided in Figure 1. The particle size distribution was relatively uniform, and an average particle diameter of ~10 nm was found. The prepared NPs exhibited mostly spherical morphology, however some of them were faceted. A more detailed image of a faceted particle produced at 20 °C is shown in Figure 2. As follows from the relevant fast Fourier transformation (FFT) pattern in Figure 2b, the particle exhibits octahedral morphology predominantly faceted by {111}-type planes. The aggregation of NPs is evident from Figure 1a,c.

The phase constitution of the produced NPs was studied using the selected area electron diffraction method (SAED) and by evaluation of the FFT patterns acquired from relevant HRTEM images (Figure 1b). Determined interplane distances of 0.485, 0.298, 0.255, and 0.212 nm correspond well with that reported for the 111, 022, 113, and 004 most-intense reflections of magnetite Fe_3_O_4_ phase (PDF No. 98-002-0596). Moreover, EELS spectroscopy was used to confirm the presence of magnetite in the samples. Quantitative EELS measurements showed that the amount of oxygen and iron in the NPs was 56.5 and 43.5 at%, respectively, which is close to chemical composition of magnetite Fe_3_O_4_.

The sharp-spotted rings in SAED/FFT patterns demonstrate a polycrystalline character of the samples and the good crystallinity of as-synthesized nanoparticles. This finding is in line with the more detailed HRTEM (high-resolution TEM) and ARTEM (atomic resolution TEM) images recorded from individual NPs prepared at 20 °C and also at 80 °C, in Figure 1 and Figure 2.

**Figure 3:**
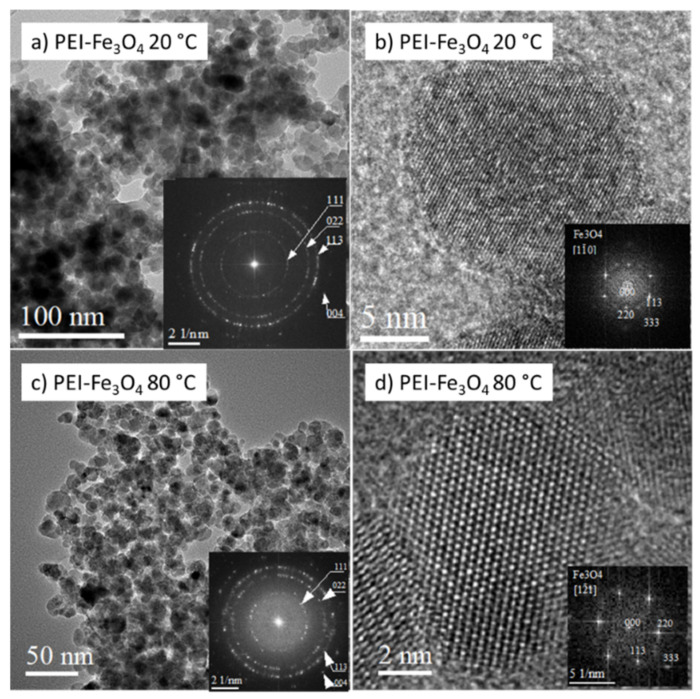
Microstructure of PEI-coated Fe_3_O_4_ NPs prepared at 20 °C (**a**,**b**) and 80 °C (**c**,**d**), respectively. Low-magnification TEM images of NPs with relevant FFT patterns in insets (**a**,**c**). HRTEM/ARTEM images of individual NPs with relevant FFT patterns in insets (**b**,**d**).

Several experimental approaches have been used to produce PEI-coated magnetic NPs, including hydrothermal, solvothermal, and co-precipitation methods [70,80,81]. However, the previously reported methods required either high temperatures, long reaction times, or several reaction steps. In our work, we have prepared PEI-coated Fe_3_O_4_ NPs at room temperature. The nanostructure of PEI-coated Fe_3_O_4_ NPs prepared at 20 °C is shown in Figure 3a. The image shows NPs of about 10 nm in size exhibiting mostly spherical morphology. However, faceted NPs, as shown in Figure 2b, are also seen in these images. The Fe_3_O_4_ NPs prepared at 80 °C and coated with PEI are shown in Figure 3c. The particle size is comparable to NPs prepared at 20 °C, confirming that the synthesis temperature can be reduced without affecting NPs’ size. Magnetite phase was confirmed by the estimation of relevant FFT patterns in both produced samples (Figure 3a,c). HRTEM and ARTEM images (Figure 3b,d) revealed that the atomic planes in NPs are well-ordered. Lattice defects, such as dislocations and stacking faults, were not detected in NPs.

**Figure 4:**
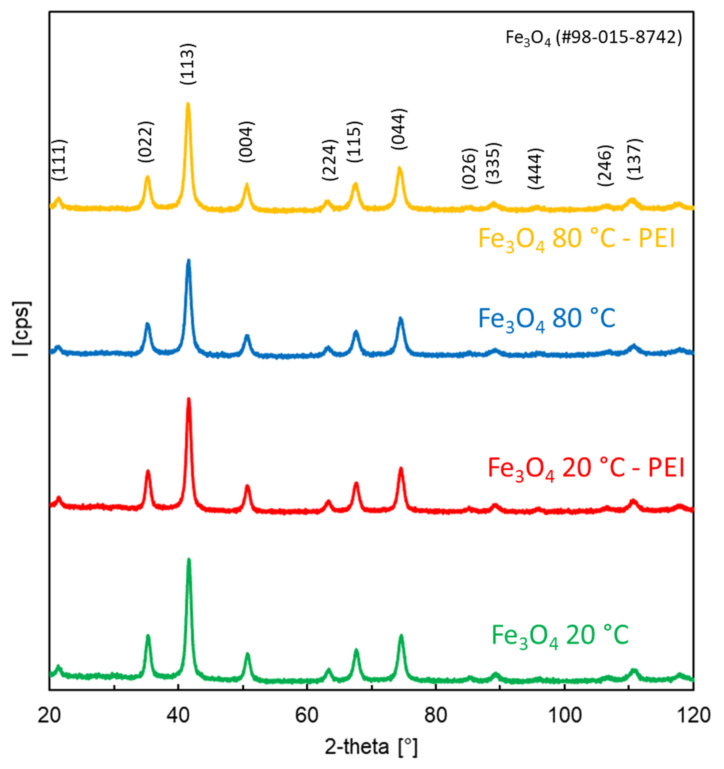
XRD patterns of Fe_3_O_4_ NPs prepared at 20 and 80 °C, and Fe_3_O_4_ NPs prepared at 20 and 80 °C and coated with PEI.

The phase constitution of the prepared NPs and NCs was also studied by room-temperature X-ray diffraction. The results are presented in Figure 4. The prepared materials were crystalline. The XRD peaks can be assigned to Fe_3_O_4_ (PDF No. 98-015-8742). The cationic polymer did not affect the crystal structure of magnetite. Furthermore, there was practically no difference in the XRD patterns of NPs and NCs prepared at 20 and 80 °C, confirming that Fe_3_O_4_ can be prepared at room temperature.

**Figure 5:**
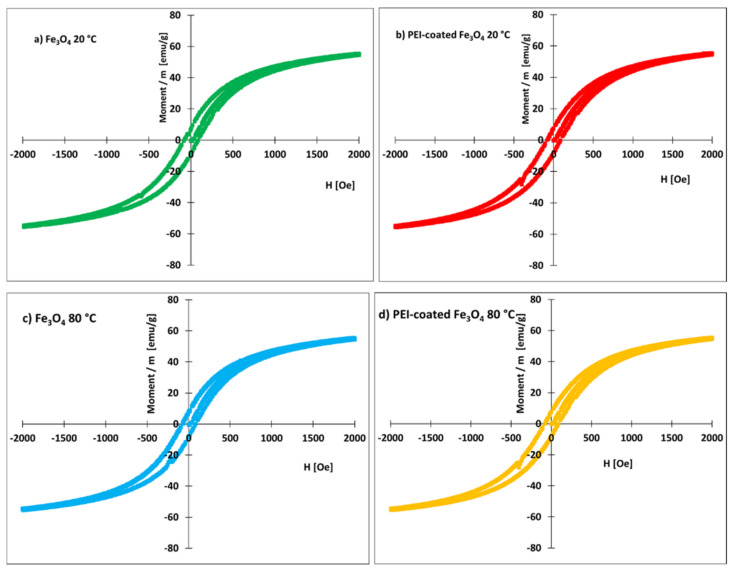
Magnetization curves of prepared nanomaterials.

The magnetization curves of the nanoparticles prepared at 20 and 80 °C are shown in Figure 5. The coated and uncoated nanoparticles had similar magnetization curves. The value of the remanent magnetization, Mr, was close to 7 emu/g. Remanent magnetization was nearly identical for both coated and uncoated nanoparticles. Thus, the organic coating does not adversely affect the value of remanent magnetization. For this reason, coated nanoparticles can be used for magnetic separation (collection) of algae from an aqueous medium.

The saturation of magnetization was close to 60 emu/g (Figure 5). This value is smaller than the previously reported 66.5 emu/g for PEI-coated Fe_3_O_4_ NPs prepared at 90 °C [82]. The smaller values are either related to the existence of nonmagnetic mass present in our samples or to nanoparticle interactions. A previous investigation [82] of the magnetic properties of PEI-coated Fe_3_O_4_ NPs suggested the existence of interacting particles, likely forming agglomerates, with a higher blocking temperature (>150 K), in which the surface spin disorder was weak and dominated by interparticle interactions. Nanoparticle agglomeration has also been observed in the present work (Figure 2a). The interparticle interactions could thus be responsible for the lower magnetic saturation.

### 3.2. Zeta Potential

**Figure 6:**
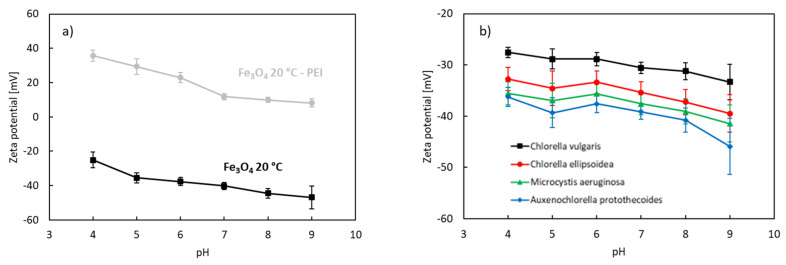
Zeta potential of naked and PEI-coated Fe3O4 NPs prepared at 20 °C (**a**) and microalgae species (**b**). Standard deviations are included.

Figure 6a shows the effect of pH on the electrokinetic zeta potential of uncoated and PEI-coated Fe_3_O_4_ nanoparticles synthesized at 20 °C. Figure 6b shows the zeta potential of the algae species tested.

The zeta potential of the uncoated nanoparticles was negative within the investigated pH range (4–9). The measured values correspond to the studies of Zhang [83], Plaza [84], Kim [85], and Savvidou [86] for magnetite nanoparticles produced by co-precipitation of iron sulfates or iron chlorides. The zeta potential increases with decreasing pH due to protonation. The isoelectric point is a point where the net electrical charge is 0. In our experiments, the isoelectric point of the uncoated magnetite nanoparticles produced by the co-precipitation method at 20 °C was estimated to be 2.0–3.0. These values are in line with the observations of Zhang et al. [83]. The zeta potential is affected not only by suspension conditions such as pH, temperature, ionic strength, and even the types of ions in the suspension, but also by particle properties such as size and concentration [87,88]. The decrease in the isoelectric point can indicate oxidation of magnetite to maghemite [89]. The final step of magnetite oxidation is maghemite [84]. Fe_3_O_4_ is not stable in the presence of oxygen, especially when stored in normal water conditions, and may undergo oxidation.

During co-precipitation of iron oxides in aqueous media (Equation (1)), surface hydroxyl groups are formed [90,91]. The hydroxyl groups are responsible for the amphoteric nature of iron oxides, leading to either positively or negatively charged surfaces depending on the pH of the solution and its ionic strength. The protonation strength values (pKa) of magnetite and its surface have been reported to be 4.4 (pKa1) and 9.0 (pKa2) [91]. The uncoated magnetic cores are prone to non-specific binding. Their stability in aqueous media is severely limited. Their colloidal stability is only achieved at extreme values and low ionic strengths. They do not have an adequate stability for most applications. To improve the stability, the bare magnetic materials are either encapsulated or coated with various chemical compounds, including surfactants and polymers [39,58,80]. The encapsulation/coating helps to stabilize the magnetic nanomaterials in aqueous solutions. The coating also helps to reduce oxidation and decrease the level of leaching of metal cations from the nanoparticle core. Furthermore, the coating process leads to an inherent inclusion of functional groups that allow further surface modification.

The surface functionalization of magnetite relies on chemical and physical forces [90]. The physical forces include electrostatic (Coulombic) interactions and van der Walls forces. Specific chemical interactions can be achieved by complexation with chelating agents. The zeta potentials of the PEI-coated nanoparticles were positive at pH 4.0–9.0 (Figure 6a). The presence of PEI thus brings a positive charge to magnetite. PEI is known for its high density of NH groups that can be easily protonated. The protonation is favored at low pH, as the concentration of H^+^ is high in acidic solutions, thereby making the surface of the nanocomposites more positively charged.

The membrane surfaces of microalgae cells are known to be terminated by functional groups –OH, –SH, and –COOH [25]. These groups can easily deprotonate. The algae species displayed a negative zeta potential within the investigated pH range (Figure 6b). Negative values decreased as pH increased due to deprotonation.

### 3.3. Magnetic Harvesting of Microalgae

**Figure 7:**
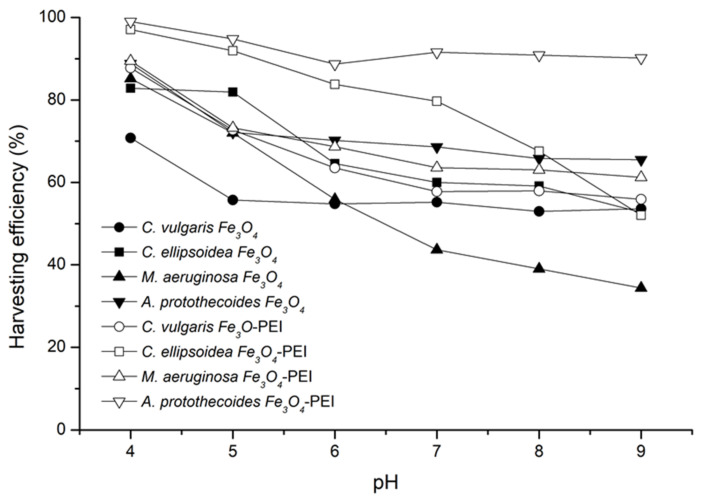
Harvesting efficiencies of uncoated and PEI-coated Fe_3_O_4_ NPs prepared at 20 °C (10 mg) at different pH levels.

Microalgae harvesting was studied at different pH levels (4–9) and different flocculant doses (5–30 mg). Harvesting efficiencies after 90 s of contact time are shown in Figure 7. Higher efficiencies, close to 100%, were obtained for PEI-coated Fe_3_O_4_ NPs. In the experiments, 10 mg of either NPs or NCs was used per testing bottle containing 50 mL of the algae suspension. The separation process was strongly pH-dependent. Harvesting efficiencies were found to decrease with increasing pH of the solution for uncoated and PEI-coated Fe_3_O_4_ NPs.

PEI-coated Fe_3_O_4_ NPs had higher harvesting efficiencies for all algae species tested within the investigated pH range. The harvesting efficiency of the uncoated magnetite at pH 8 was 39–53%, while for the PEI-coated magnetite it was 58–90%, respectively. Lowering the pH from 8.0 to 4.0 resulted in a significant increase in harvesting efficiencies. *A. protothecoides* and *C. ellipsoidea* reached harvesting efficiencies of 99% at pH 4 using PEI-coated Fe_3_O_4_ NPs as magnetic flocculants.

**Figure 8:**
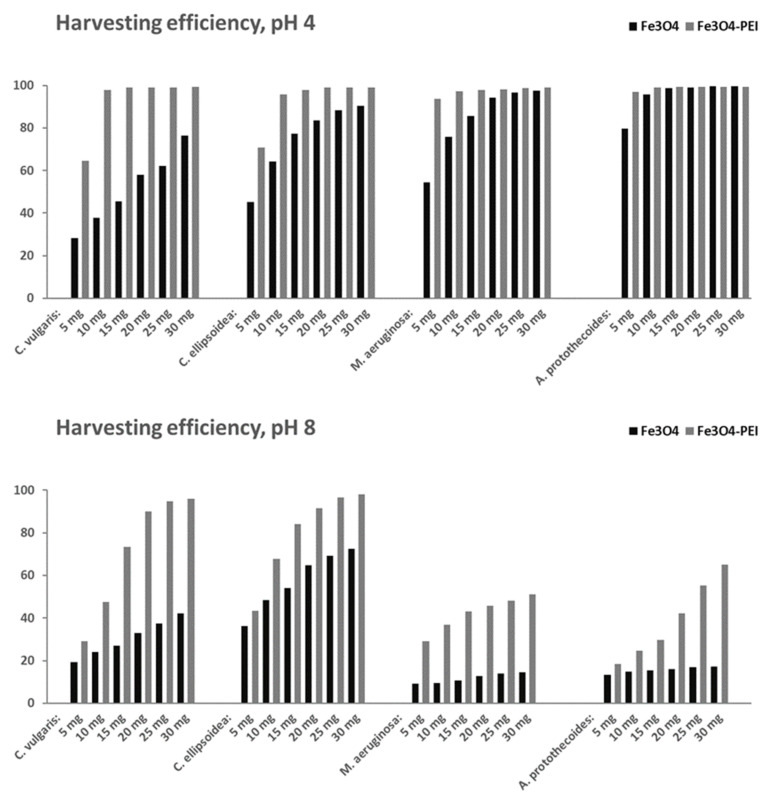
Harvesting efficiencies (in %) of different doses of uncoated and PEI-coated Fe_3_O_4_ NPs prepared at 20 °C, at pH 4 (**top**) and 8 (**bottom**).

We also tested several different doses of uncoated magnetite and PEI-coated magnetite. In all cases, a higher dose resulted in higher efficiencies for both coated and uncoated magnetite. The results are presented in Figure 8. To reach a minimum efficiency of 90% at pH 4, 30 mg of Fe_3_O_4_ but less than 10 mg of Fe_3_O_4_-PEI were needed for *C. ellipsoidea*. At pH 8, 20 mg of Fe_3_O_4_-PEI caused a harvesting efficiency greater than 90%. The results again show that higher harvesting efficiencies were achieved in an acidic environment. To reach 98–99% harvesting efficiency, the optimal dosage at pH 4 is 10 mg of PEI-coated NP for *C. vulgaris*, *C. ellipsoidea*, *M. aeruginosa,* and *A. protothecoides*. For uncoated NPs, this level of harvesting efficiency was reached for only two algae species (*C. ellipsoidea* with the dose of 20 mg, and *A. protothecoides* with 15 mg). At pH 8, *C. ellipsoidea* reached 98% harvesting efficiency at the dose of PEI-coated NPs equal to 30 mg. This result shows that this algae species can be harvested in neutral conditions.

Harvesting efficiencies of the investigated green algae and cyanobacteria are compared in Table 2. Our results show comparable harvesting efficiencies to previously reported results [65,68,70,92,93]. The difference is minor and is attributable to either the utilization of non-specified *Chlorella* species, higher temperature during co-precipitation, higher pH of the solution (pH 7–8), or longer time used for the separation (up to 30 min). It can be observed that the decreased co-precipitation temperature causes a decrease in the harvesting efficiency. Hu et al. reached 97% of the harvest efficiency of *C. vulgaris* with PEI-coated NPs synthesized by the co-precipitation method at 80 °C with the dosage of 20 mg L^−1^ at pH 9 in 2 min [70]. In our study, 30 mg of PEI-coated NPs reached 96% of the harvesting efficiency at pH 8. On the other hand, only 80% of the harvesting efficiency with PEI-coated NPs was achieved in the study by Wang et al. [68] for *Microcystis aeruginosa*. In our study, we reached more than 93% of harvesting efficiencies with 5 mg of PEI-coated NPs. Therefore, it can be concluded that the harvesting efficiency for the PEI-coated magnetite NPs is comparable to previous studies.

### 3.4. Adsorption Isotherms

**Figure 9:**
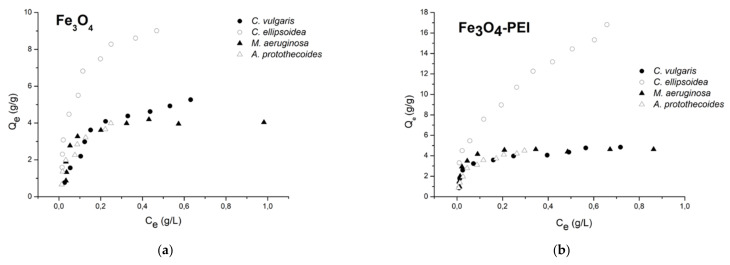
Adsorption isotherms for the sorption systems of tested algae and (**a**) non-coated magnetite and (**b**) PEI-coated magnetite prepared at 20 °C, pH 7.

A wide variety of adsorption isotherm models have been studied in the literature. The models can be classified as follows: (1) irreversible isotherms and one-parameter isotherms (e.g., Henry isotherm), (2) two-parameter isotherms (e.g., Langmuir, Freundlich, and Dubinin–Radushkevich, which are the most used), (3) three-parameter isotherms (e.g., Redlich–Peterson), and (4) more than three-parameter isotherms [94]. The adsorption isotherms illustrate the equilibrium relationship between the adsorption capacity (the equilibrium-adsorbed amounts) and the equilibrium concentration in the solution for a constant equilibrium pH and temperature of the solution [95]. In our work, the equilibrium between harvested microalgal cells and their concentration in the supernatant has been studied by the Langmuir and Freundlich isotherms. Experimental data for both uncoated and PEI-coated magnetite are displayed in Figure 9. Parameters estimated from linear and nonlinear Langmuir and Freundlich models are summarized in Table 3.

According to the data presented in Table 3, a better fit was found for the Langmuir than the Freundlich model, except for *C. ellipsoidea* and Fe_3_O_4_-PEI. This result agrees well with [35]. When comparing the utilization of linear and nonlinear models, we obtained better correlation in linear models for the Langmuir models than in the nonlinear models; however, when the Freundlich models were used, the nonlinear extrapolation was more accurate.

At pH 7, although the PEI coating brings an extra positive charge to the magnetite particles, the maximum adsorption capacity was lower. For example, for *C. vulgaris,* it was more than 26% lower (4.932 compared to 6.700 g g^−1^). The highest adsorption capacity was obtained in the case of coated magnetite and *Chlorella ellipsoidea* (18.612 g g^−1^), while the lowest adsorption capacity was obtained for noncoated magnetite and *Microcystis aeruginosa* (4.369 g g^−1^).

Studies of adsorption kinetics play an important role in identifying the required equilibration time, the optimal contact time, and the mechanism of the adsorption process [60]. The kinetic aspects of adsorption have not yet been studied in detail. However, it has been observed in our study that the sorption process of all algae species occurs rapidly, as the settling processes take only approximately 15–30 s.

### 3.5. Adsorption Mechanism

There exist four steps associated with materials’ transport during adsorption [94]. The first stage is solution phase transport, known as “bulk transport”. The bulk transport can occur instantaneously after the adsorbent is transported into the adsorbate solution. As such, its contribution to the overall rate of adsorption is negligible. The second stage is “film diffusion”. In the second stage, the adsorbate molecules are transferred from the bulk liquid phase to the adsorbent’s external surface through the hydrodynamic boundary layer or film. The third stage—interparticle diffusion—involves the diffusion of the molecules from the exterior into the pores of the adsorbent, along pore-wall surfaces, or both. The diffusion stage occurs slowly and may be rate-limiting. The last stage is an adsorptive attachment. It often occurs quickly, and therefore, it is not considered to be significant for the adsorption kinetics [94].

There also exist several possible mechanisms in algae harvesting with the uncoated and coated magnetite, including charge neutralization, patching, or adsorption bridging. In charge neutralization, the net charges of the microalgae particles are cancelled by adsorbing an equivalent number of opposite charges. An oppositely charged flocculant added into the culture medium increases the ionic strengths of the medium and the concentration of counter ions, but decreases the particle charges and the zeta potential. It allows the formation of van der Waals force of attraction to encourage initial aggregation. The electrostatic patch (patching) mechanism is the phenomenon in which a charged polymer binds to a particle with opposite charge. The polymer locally reverses the charge of the particle surface, resulting in patches of opposite charge on the particle surface. Patching occurs when unevenly distributed surface charges are incompletely neutralized. After that, particles may connect with each other through patches of opposite charge. In general, adsorption bridging occurs when long-chain polymers with high molecular weight and low charge density have been adsorbed on particles in such a way that long loops and tails extending or stretching into solution far beyond the electrical double-layer polymers or charged colloids simultaneously bind to the surface of two different particles to form a bridge between these particles [96,97,98,99,100,101,102].

**Figure 10:**
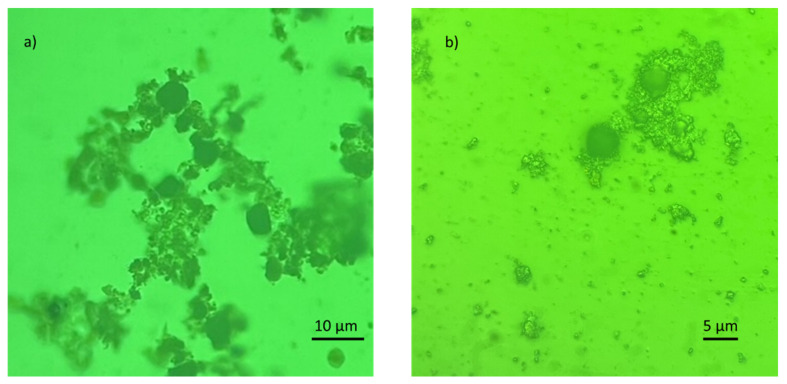
*Auxenochlorella protothecoides* cells with adsorbed Fe_3_O_4_-PEI nanocomposites at different magnifications (**a**,**b**).

The electrostatic interaction of algae and coated nanoparticles was important as the zeta potentials in the studied pH range were opposite. However, electrostatic forces may have not been sufficient when the non-coated magnetite was used, as the zeta potential of both species lay in the negative region (Figure 6). Savvidou et al. [86] suggested that Fe_3_O_4_ particles can be attached to microalgal cells by hydrogen bonding. Due to significant protonation of magnetite particles under acidic conditions, the chemical species of the hydrogen bond donor OH_2_^+^ can be formed in Fe_3_O_4_ and interact with the hydrogen bond acceptor groups present in *C. vulgaris* cells, such as amino or carboxy groups. The authors of [64] suggested that the principal mechanism of the algae harvesting process (*C. vulgaris* and tailor-made magnetic nanoparticles) was bridging. Furthermore, some authors [60] used an extended Derjaguin–Landau–Verwey–Overbeek (EDLVO) theory to demonstrate the contributions of the surface properties of membranes or flocculants to colloidal interactions. The EDLVO theory may be used to reveal the principles of interaction between the magnetic nanoparticles and algae cells. Although the electrostatic interaction is described by the net characteristic of all charged groups on the surface of microalgae cells and the magnetic surface of NP, it might appear in sophisticated bilayer microdomains [60]. This fact is confirmed by microscopy images of the system after adhesion, which show an agglomerated Fe_3_O_4_ structure that covers only a part of the microalgae cell wall and is heterogeneously distributed, leaving parts of the cell wall surface free (Figure 10). Furthermore, additional facts must be considered. The aggregation of superparamagnetic magnetite nanoparticles may also be affected by their magnetic properties, which are in competition with the repulsive forces of van der Waals and electrostatic interactions. The final agglomeration influences the mobility and reactivity of the nanoparticles and depends on several factors, that include the pH of the solution, the ionic strength, and the presence of organic matter. In biological experiments where nanoparticles are suspended in solution, the composition, density, viscosity, and physiochemical characteristics of the cell culture medium must also be considered, as they can interfere with the stability and aggregation of magnetic NPs [87,103].

The biochemical composition of the algae cell surface differs between species and is variable within a species, for example, exponential versus stationary phase cultures. Furthermore, microalgae often excrete significant amounts of organic matter, consisting of polysaccharides and proteins in growth medium, which can promote or inhibit floc formation [86]. Furthermore, chelating metal cations [104] can play an important role in the interaction with the flocculant as they are attached to the cell walls [97]. Another point is that algae are typically cultivated axenically only when storing the cultures, and afterward when the experiment is realized, cultivation is not sterile. The presence of bacteria that enter the system may also produce different kinds of extracellular polymeric substances, which can affect both the magnetic nanoparticles and the flocculation behavior of algae cells.

## 4. Conclusions

In the present work, magnetic harvesting of *Chlorella vulgaris, Chlorella ellipsoidea, Microcystis aeruginosa,* and *Auxenochlorella protothecoides* has been studied. Microalgae were obtained as sterile cultures from the algae collection of the University of Göttingen, Germany. The prepared microalgae were harvested with magnetite (Fe_3_O_4_) nanoparticles. The nanoparticles were prepared by controlled co-precipitation of Fe^2+^ and Fe^3+^ cations in ammonia at room temperature. Subsequently, the prepared Fe_3_O_4_ were coated with polyethyleneimine (PEI). The prepared materials were characterized by TEM, magnetometry, and zeta potential measurements. The following conclusions can be made:

1. The prepared NPs were spherical. The particle size distribution was relatively uniform and an average particle diameter of ~10 nm was found. The NPs prepared at 20 °C were smaller. However, the difference in nanoparticle diameter between materials prepared at 20 and 80 °C was not significant. The crystal structure of magnetite was confirmed by electron diffraction.
2. The zeta potential of the uncoated nanoparticles was negative within the investigated pH range (4–9). The zeta potentials of the PEI-coated nanoparticles were positive at pH 4–9. The presence of PEI thus brings a positive charge to magnetite.
3. The algae species displayed a negative zeta potential within the investigated pH range. Negative values decreased as pH increased due to deprotonation.
4. Microalgae harvesting was studied at different pH levels and different flocculant doses. Higher efficiencies, close to 100%, were obtained for PEI-coated Fe_3_O_4_ NPs.
5. The adsorption of magnetic flocculants on harvested microalgal cells has been studied by Langmuir and Freundlich isotherms. A better fit was found for the Langmuir isotherm, indicating a monolayer adsorption.

The results show that the synthesis of magnetic nanoparticles at 20 °C is feasible. The nanoparticles have comparable characteristics (particle size, microstructure, harvesting efficiency) to NPs prepared at 80 °C. The harvesting efficiencies can be significantly enhanced by PEI coating, as the polymer increases the positive electrical charge of the nanoparticles. High efficiencies, close to 100%, were obtained for PEI-coated Fe_3_O_4_ NPs at pH 4. Relatively high efficiencies can be obtained at pH 8, which makes the separation process feasible in neutral conditions. Furthermore, the magnetic harvesting with the nanoparticles synthesized at room temperature is applicable to both green algae and cyanobacteria, making the process attractive for industrial use.

## Figures and Tables

**Table 1:** Langmuir and Freundlich adsorption isotherms.

	Nonlinear Form	Plot	Linear Form	Plot
Langmuir	$Q_{e}=\frac{Q_{m}K_{L}C_{e}}{1+K_{L}C_{e}}$	$C_{e}\;vs.\;Q_{e}$	$\frac{C_{e}}{Q_{e}}=\frac{1}{Q_{m}}C_{e}+\frac{1}{K_{L}Q_{m}}$	$C_{e}\;vs.\;\frac{C_{e}}{Q_{e}}$
Freundlich	$Q_{e}=K_{F}C_{e}^{\frac{1}{n_{F}}}$	$C_{e}\;vs.\;Q_{e}$	$\ln\left(Q_{e}\right)=\ln\left(K_{F}\right)+\frac{1}{n_{F}}\ln\left(C_{e}\right)$	$\ln\left(C_{e}\right)\;vs.\;\ln\left(Q_{e}\right)$ or $\log\left(C_{e}\right)\;vs.\;\log\left(Q_{e}\right)$

In these equations: *Q_m_* is the maximum adsorption capacity (g g^−1^), *K_L_* is the Langmuir adsorption constant (L g^−1^), *K_F_* is the Freundlich constant related to the adsorption capacity (g g^−1^), and *n_F_* is the Freundlich heterogeneity factor of the adsorption sites (dimensionless).

**Table 2:** Harvesting efficiencies of green algae and cyanobacteria.

Microalgae/ Cyanobacteria	Algae DCW (g L^−1^)	NPs Type	Dosage g Floculant/g of Dry Algae	pH	Contact Time (s)	Harvesting Efficiency (%)	Reference
*M. aeruginosa*	1.788	Fe_3_O_4_	0.112	4	90	85.2	This study
*M. aeruginosa*	n.a.	Fe_3_O_4_	0.58	3	300	99	[65]
*M. aeruginosa*	1.788	Fe_3_O_4_-PEI	0.112	4	90	89.4	This study
*M. aeruginosa*	n.a.	Fe_3_O_4_-PEI	0.14–0.18	3	70–95	93-97	[68]
*C. ellipsoidea*	1.128	Fe_3_O_4_	0.177	4	90	82.9	This study
*C. ellipsoidea*	n.a.	Fe_3_O_4_	0.3	4	60	90	[92]
*C. ellipsoidea*	1.128	Fe_3_O_4_-PEI	0.177	4	90	97.0	This study
*C. ellipsoidea*	0.75	Fe_3_O_4_-PEI	0.026	4	120	98	[70]
*C. vulgaris*	1.683	Fe_3_O_4_	0.119	4	90	70.8	This study
*C. vulgaris*	n.a.	Fe_3_O_4_	2.0	4	120	>70	[93]
*C. vulgaris*	1.683	Fe_3_O_4_-PEI	0.119	4	90	87.8	This study
*A. protothecoides*	0.746	Fe_3_O_4_	0.268	4	90	88.8	This study
*A. protothecoides*	0.746	Fe_3_O_4_-PEI	0.268	4	90	99.0	This study

**Table 3:** Adsorption isotherm parameters for the studied algae-NPs species at 25 °C. Microalgal growth stage: 14 days, pH 7. Explanation of abbreviations: Initial algae concentration (*C*_0_). Maximum adsorption capacity (*Q_m_*). Langmuir adsorption constant (*K_L_*). Freundlich adsorption constant (*K_F_*). Freundlich heterogeneity factor of adsorption sites (*n_F_*). Correlation coefficient (*R*). Constant separation factor (*R_L_*). Chi-squared test (χ^2^).

Microalgae Species	*Chlorella vulgaris*	*Chlorella ellipsoidea*	*Microcystis aeruginosa*	*Auxenochlorella protothecoides*	*Chlorella vulgaris*	*Chlorella ellipsoidea*	*Microcystis aeruginosa*	*Auxenochlorella protothecoides*
sorbent	Fe_3_O_4_	Fe_3_O_4_	Fe_3_O_4_	Fe_3_O_4_	Fe_3_O_4_-PEI	Fe_3_O_4_-PEI	Fe_3_O_4_-PEI	Fe_3_O_4_-PEI
dose (mg)	10.0	5.0	10.0	5.0	10.0	2.5	10.0	5.0
model	Langmuir linear				Langmuir linear			
*C*_0_ (g L^−1^)	1.6848	1.4979	1.7894	0.7463	1.6848	1.4979	1.7882	0.7463
*Q_m_* (g g^−1^)	6.700	10.222	4.369	4.874	4.932	18.612	4.735	4.871
*K_L_* (L g^−1^)	5.666	15.310	17.954	15.153	23.306	7.070	51.257	26.088
R^2^	0.980	0.994	0.985	0.962	0.990	0.947	0.998	0.993
model	Freundlich linear				Freundlich linear			
*C*_0_ (g L^−1^)	1.6848	1.4979	1.7894	0.7463	1.6848	1.4979	1.7882	0.7463
*K_F_* (g g^−1^)	8.154	15.078	5.698	8.763	5.830	18.252	5.926	7.956
1/*n_F_*	0.577	0.454	0.350	0.511	0.303	0.382	0.269	0.405
R^2^	0.905	0.909	0.637	0.862	0.823	0.986	0.709	0.953
model	Langmuir nonlinear				Langmuir nonlinear			
*C*_0_ (g L^−1^)	1.6848	1.4979	1.7894	0.7463	1.6848	1.4979	1.7882	0.7463
*Q_m_* (g g^−1^)	6.519	10.105	4.548	4.683	4.601	19.523	4.843	4.753
*K_L_* (L g^−1^)	6.364	16.034	17.800	17.254	38.964	5.609	49.245	28.084
R^2^	0.972	0.984	0.886	0.947	0.938	0.925	0.945	0.980
χ^2^	0.0653	0.1178	0.2063	0.0692	0.1067	0.4899	0.1055	0.0309
*R_L_*	0.0853	0.0399	0.0304	0.0720	0.0150	0.1063	0.0112	0.0455
model	Freundlich nonlinear				Freundlich nonlinear			
*C*_0_ (g L^−1^)	1.6848	1.4979	1.7894	0.7463	1.6848	1.4979	1.7882	0.7463
*K_F_* (g g^−1^)	6.757	12.908	4.720	28.084	5.369	19.268	5.319	6.986
1/*n_F_*	0.434	0.370	0.255	0.426	0.245	0.424	0.199	0.345
R^2^	0.912	0.934	0.699	0.935	0.914	0.990	0.755	0.964
χ^2^	0.2027	0.0491	0.4497	0.0846	0.1467	0.0335	0.4702	0.0573

## Data Availability

Data are available from the corresponding author upon request.
